# Oral feeding challenges of infants of diabetic mothers

**DOI:** 10.3389/fped.2024.1459197

**Published:** 2024-09-09

**Authors:** Leslie-Anne J. Dietrich

**Affiliations:** ^1^ Pediatrics, Division of Neonatology, University of Texas Health San Antonio, San Antonio, TX, United States; ^2^ University Health System, San Antonio, TX, United States

**Keywords:** diabetes, infants of diabetic mothers, feeding difficulty, oral feeding, infant behavior

## Abstract

**Objective:**

The presence of diabetes before or during pregnancy can increase perinatal mortality and morbidities. It is well known an infant of a diabetic mother (IDM) may experience complications such as macrosomia, hypoglycemia, respiratory distress syndrome, cardiac anomalies, and other abnormalities of organogenesis. Medical providers including physicians, nurses, and speech therapists have experienced challenges with helping IDMs orally feed. Challenges with oral feeding can lead to prolonged hospital stays and placement of supplemental feeding devices. The etiology of an IDM's oral feeding delays is not well understood and does not necessarily affect all infants.

**Study design:**

This descriptive review explores what is known about potential contributing factors to feeding difficulty in IDMs, including differences in infant behavior and swallowing mechanics.

**Results:**

Some IDMs are unable to maintain active alert states and have decreased autonomic regulation and motor control. Studies of sucking and swallowing demonstrate reduced sucking pressure, fewer sucking bursts, and slowing of esophageal sphincter function.

**Conclusion:**

The increasing prevalence of diabetes during pregnancy makes further investigations into the characteristics and trajectories of state, behavior, and oral feeding of IDMs imperative.

## Introduction

During pregnancy women may be affected by type 1 diabetes, type 2 diabetes, or gestational diabetes mellitus. Gestational diabetes mellitus (GDM) is a form of diabetes that develops during the pregnancy, and pregestational diabetes is when type 1 or type 2 diabetes develops before pregnancy. When diabetes affects a woman during pregnancy, the fetus is at risk for multiple complications (1). The majority of fetal and neonatal challenges are the result of hyperglycemic and hyperinsulinemic states in the infant, secondary to maternal hyperglycemia. Complications often seen after birth include macrosomia, hypoglycemia, hypertrophic cardiomyopathy, and respiratory distress syndrome. Infants may be affected with critical congenital heart disease (e.g., transposition of the great arteries), neural tube defects, kidney anomalies, skeletal defects, or gastrointestinal malformations (e.g., imperforate anus, intestinal atresias, microcolon). The risk for congenital anomalies is higher among infants born to mothers with pregestational diabetes compared to those born to mothers with gestational diabetes; the risk is 3–5 times higher when the condition is poorly controlled (2).

According to the CDC in 2019, 11.3% of the general population, that is 37.3 million adults were living with diabetes in the United States (3). The Southern and Eastern regions have the highest rates of adults ≥20 years of age diagnosed with diabetes. Estimates do not delineate between type 1 and type 2 diabetes, and likely represent the more prevalent type 2 diabetes. Similarly, the prevalence of pregestational diabetes has doubled in the last 3 decades (4). GDM occurs in 2%–10% of pregnancies and has increased 30% from 2016 to 2020 in the United States (2).

One poorly understood complication for infants of diabetic mothers (IDMs) is oral feeding difficulties. Some infants have medical complications, such as neurologic or cardiorespiratory problems, that can delay time to oral feeding. Some IDMs struggle with learning to orally feed even when these conditions are not present or have long since resolved (5). The exact incidence of oral feeding difficulties in IDMs is not known. A study from the 1970s documented poor feeding in 37% of a cohort of 147 IDMs with varying gestational ages and complications (6). Compared to infants born to mothers without diabetes, IDMs seem to have difficulty engaging while feeding, slow development of interest to orally feed, and low tone. This difficulty with oral feeding may go overlooked and poorly emphasized as a potential problem for IDMs. Since little is known about which IDMs are at risk, predicting this complication is challenging. This article is a review of the current evidence concerning oral feeding of IDMs, as well as the behavior that has the potential to impact their oral feeding skills.

## Behavior of IDMs

An important factor in an infant's ability to feed is their capacity to maintain an ideal state (7). Brazelton classified infant states into 6 categories: deep sleep, light sleep, drowsy, alert, active alert and crying (8). Premature infants tend to spend more time in the deep sleep, light sleep, and drowsy states with less time in the alert and active alert states. As gestational age (GA) increases infants typically transition more easily between states, are better able to maintain a state, and spend more time in the alert and active alert states. The alert state is typically the ideal state for feeding to occur (7). Successful feeding requires the infant to both maintain an alert state, and possess sufficient endurance and oral-motor tone.

Providers frequently refer to the behavior of IDMs to families as “immature” or “preterm” in nature. However, is it truly the same behavior as a preterm infant with the ultimate expectation the baby will mature to normal state transitions as they get older? How long will this transition to maturation of state regulation be expected to take?

Studies in the 1980s to 1990s used the Neonatal Behavioral Assessment Scale (NBAS) to demonstrate that IDMs' ability to maintain alert and active alert states appears to be impaired (10–12). In the late 1960s to early 1970s Berry Brazelton et al. (9) studied infant behavior and developed the NBAS. The NBAS is designed to evaluate infant behavior from birth through 2 months of age. It consists of 28 behavioral items and 20 items to assess the neurological status. The NBAS was created with a notion that the infant is inherently programmed to interact and exhibit social behavior with the caregiver. Although portions of the exam are observation of reflexes, much of the exam is based upon infant responses to interactions with the examiner.

The NBAS assesses the infant's ability to adapt and self-regulate (9). The 4 domains of neurobehavioral functioning evaluated during the NBAS are autonomic/physiological regulation, motor organization, state organization and regulation, and attention/social interaction. Typically, the infant will demonstrate an evolution of integrating all 4 domains over the first 2 months of life. The infant normally begins by organizing autonomic/physiologic regulation as seen with thermoregulation, breathing stability, and controlling the number of tremors and startles. Next, the infant controls motor behavior with improvements in muscle tone and harnessing integration of movements. The third task for the infant is learning state regulation. The infant will exhibit predictable sleep and wake cycles, as well as demonstrate ways to self-regulate (e.g., hands to mouth, crying and consolable). Lastly, the infant's interactive behaviors mature. The infant has prolonged periods of alertness, maintains engagement with visual and auditory stimuli, and searches for and participates in caregiver interactions.

**Figure 1:**
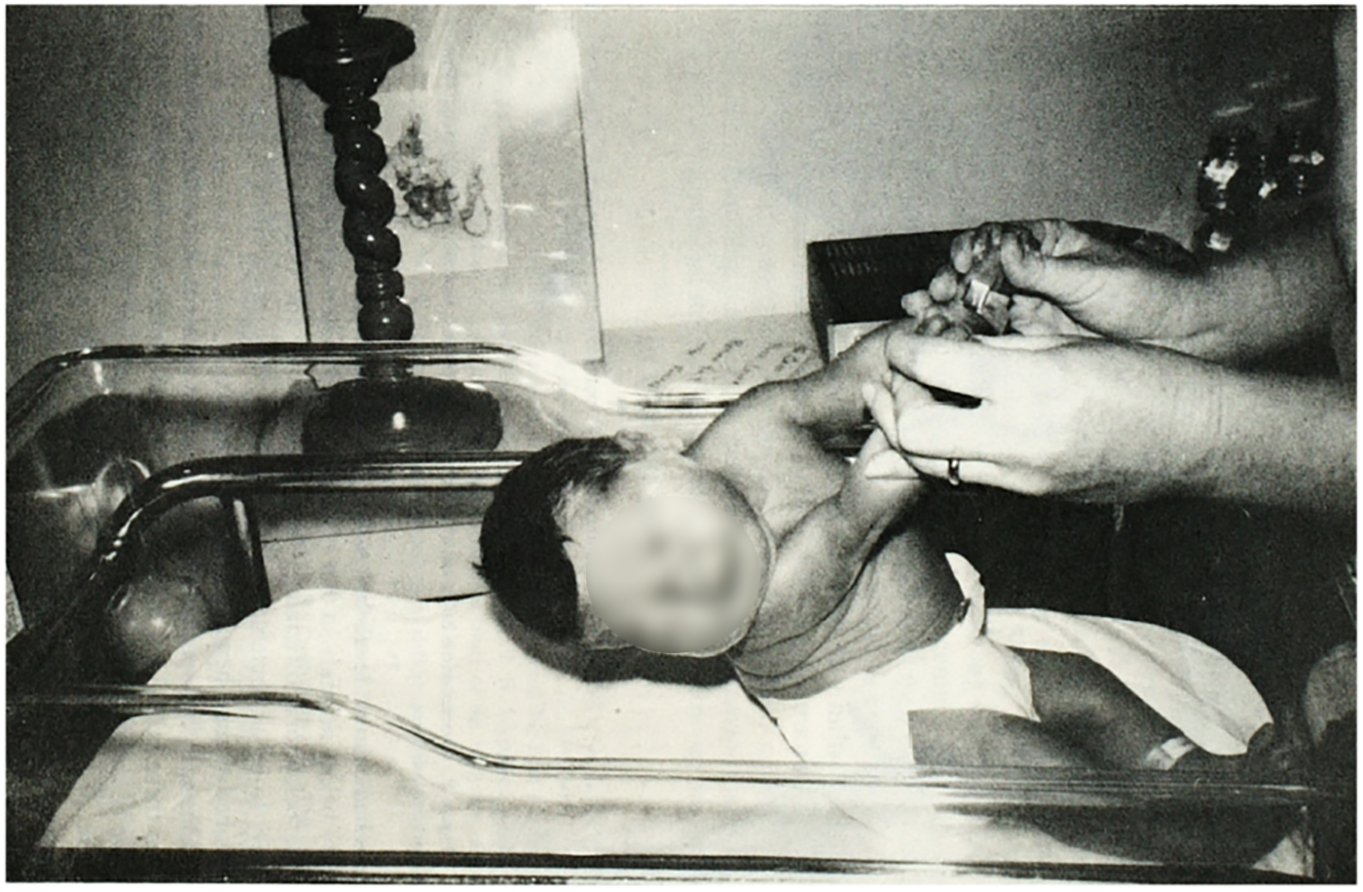
Pull-to-sit maneuver performed with a 3-day old infant of a mother with diabetes. Note the poor head control. Reproduced from Yogman MW, Cole, P, Heidelise A, and Lester BM, Behavior of newborns of diabetic mothers. Infant Behavior and Dev. 1982; 5: 331–340 © Elsevier.

In 1982 Yogman et al. (10) used the NBAS to compare behavior of 10 infants of mothers with diabetes vs. 10 infants of mothers without diabetes. The GA at birth for IDM vs. the control group was approximately 37 vs. 39 weeks GA. When the infants were examined with the NBAS on days of life 3, 4, and 7 IDMs scored lower in areas of visual and auditory orientation, motor performance, and autonomic stability. IDMs performed the lowest for motor items on day of life 7. Specifically, the IDM newborns exhibited poor head control with pull-to-sit motion and had difficulty visually tracking a human face with or without the presence of that person's voice (Figure 1). IDMs had more difficulty maintaining periods of alertness. While severity of maternal diabetes varied, the researchers noted all mothers were closely monitored and well controlled during their pregnancy.

In 1990, Rizzo et al. (11) evaluated infant behavior using the NBAS comparing infants of 73 pregestational diabetic mothers, 112 GDM mothers, and 24 nondiabetic mothers. Infants born <37 weeks GA were assessed with the NBAS at 40 weeks post-menstrual age (PMA) and term infants were assessed at 2–3 days of life. Of note, in the population of mothers with diabetes, the hemoglobin A1C (HbA1C) levels were higher for women with pregestational diabetes and poor control prior to enrolling in the obstetrics clinic compared with GDM and nondiabetic mothers. The mean GA of infants born to pregestational and gestational diabetic groups were significantly lower than the nondiabetic group, 38.2 ± 0.2 and 38.6 ± 0.2 vs. 39.5 ± 0.4 weeks. However, the incidence of preterm delivery did not differ between groups. Birthweight was higher in the two diabetic groups. The occurrence of hypoglycemia in infants born to mothers with pregestational diabetes was significantly higher than the nondiabetic and gestational diabetic mothers. After controlling for prematurity, there were significant differences in behavior depending upon the 2nd and 3rd trimester HbA1C and fasting plasma glucose levels. When maternal glucose and HbA1C levels were higher, the physiologic and motor control responses from IDMs on the NBAS were slower, respectively.

In 1999 Pressler et al. (12) observed differences in behavior between 40 newborns born at 37–42 weeks GA to mothers with diabetes during pregnancy vs. 40 control newborns 37–42 weeks GA of non-diabetic mothers. Infants' behaviors were examined at 12–24 h and 36–48 h of life using the NBAS. Motor processes and reflex functioning were significantly lower for the IDM group than the control group. The IDMs also had decreased muscle tone, reduced arousal, and “sluggish” behavior. Motor processing, autonomic stability, reflex functioning, and modal performance improved at the 36–48-h assessment when compared to the 12–24-h assessment, but remained lower than the control group. The groups were not followed further, so the time required for IDMs to perform at the level of the control group—if ever—is unknown.

However, in 1996, Botet et al. (13) used the NBAS on day of life 3 to assess 15 infants born to mothers with GDM, 3 infants born to mothers with type 1 diabetes, and 32 infants born to healthy mothers; they found no difference in behavior. It is important to note, that all patients in the study whose mothers had diabetes had good control with a max HbA1C level of 7.4 g/L. Therefore, adequate control of diabetes may be enough to mitigate the effects on an infant's behavior.

## Oral feeding difficulties in IDMs

Medical providers have long observed that IDMs are at high risk for a prolonged length of stay due to delays in learning to feed and poor interest. The incidence of feeding difficulties in the population of IDMs remains understudied. Minimal research has been done to describe the challenges this group of infants may face and how to best support them.

In 2006, Bromiker et al. (5) from Israel were the first to characterize the details of sucking patterns in IDMs. They compared 31 term infants of mothers with diet controlled GDM (diet-GDM group), 16 infants of mothers with insulin controlled GDM (insulin-GDM group), and 55 term infants whose mothers did not have diabetes (control group). All infants had been cared for in the well-baby nursery. Compared to the control group, the insulin-GDM group had a higher incidence of birth via C-section and were born 1 week earlier (38 weeks vs. 39 weeks GA). Both the insulin-GDM and diet-GDM infants had hypoglycemia after birth and the infants in the control group did not. Sucking patterns for the first 5 min of the feed were observed on day of life 3. The insulin-GDM group had approximately 5.2 fewer sucking bursts and 42 fewer sucks per 5-minute interval. No significant differences were found between the diet-GDM and control groups in total number of sucks, total numbers of bursts, average number of sucks per burst, time between bursts, average suck width and average maximum suck pressure. This study is limited by the fact that infants' sucking characteristics were measured at a single feed and for only the first few minutes of that feed. Studies that focus on an entire feeding session are needed, as are studies that follow overall feeding progression of IDMs in the first few weeks to months of life.

Malkar et al. (14) studied manometry testing of 20 IDMs with dysphagia and 10 control infants at approximately 39–42 weeks PMA. The sample of IDMs had a mean GA 34.2 ± 1.2 weeks, and 55% of the group was large for gestational age. Compared with controls, the IDM group had longer periods of upper esophageal sphincter relaxation and longer time to initiate esophageal peristalsis. IDMs had a lower frequency of deglutination apnea than the control infants. Although the lower esophageal sphincter nadir pressure was similar between groups, the IDM group spent more time at the LES nadir. Despite the delayed response times of the upper and lower esophageal sphincters, the remaining parameters, such as swallow propagation, did not differ significantly between groups. Nine (45%) of the 20 IDMs would go on to need gastrostomy tube (GT) placement at hospital discharge and 6 required oxygen at discharge. Additional comorbidities were not described by the authors. The authors propose a vagal-type neuropathy as the cause for differences in esophageal function.

Viswanathan et al. (15) examined the role of reflux in oral feeding difficulties of IDMs. Acid reflux was quantified using impedance-pH monitoring at approximately 42 weeks PMA in 50 IDMs with a GA at birth of 30–38 weeks. While 40% of the group had impedance-pH monitoring consistent with reflux, outcomes of oral feeding success, GT placement rates, and length of stay were not altered by presence of reflux nor proton pump inhibitor medication. The infants exhibiting pathologic reflux had a lower birth weight (2,356.0 ± 1,369.1 vs. 2,660.0 ± 1,225.8 g) and weight at impedance-pH testing (4,136.7 ± 1,063.1 vs. 3,519.5 ± 916.1 g) than those without reflux. Otherwise, there was no difference between groups including GA at birth, PMA at time of impedance-pH testing, Apgar scores, hypoglycemia, or need for respiratory support. Need for GT placement was 40% (20/50) of the cohort and the GT was placed at approximately 45–57 weeks.

Many describe IDMs as having little interest in oral feeding and not exhibiting typical infant hunger cues. Some speculate that high fat mass may affect an infant's hunger drive secondarily from the hormones secreted (16). Studies have shown large for gestational age (LGA) infants and IDMs have high leptin (satiety hormone) and low ghrelin (hunger hormone) levels in umbilical cord blood (17, 18). This combination would lead to one having less of a desire to eat. Short KR et al. (19) demonstrated term IDMs have a reduced resting energy expenditure. Viswanathan et al. (16) found of those infants admitted to the NICU requiring more enteral tube feeds and feeding less orally at 37 weeks PMA had a higher percentage of fat mass and higher fat mass z-score compared to the control group. Seven of the 16 infants with increased reliance on tube feeds had mothers with diabetes and were LGA. Five infants with decreased oral feeding required GT placement prior to discharge.

An additional barrier providers encounter is determining the optimal nutritional intake for IDMs. Being LGA is a known characteristic of many IDMs and therefore, diet calculations that are weight-based will lead to a daily caloric intake and milk volumes higher than are typical for appropriate for gestational age infants. Consequences from high milk volumes and caloric intake include but are not limited to reflux, intestinal discomfort, unnecessary pressures imposed upon the infant to complete large feeds, and potentially the development of metabolic disease in adulthood. Consideration should be given to tailoring nutritional plans to meet the needs of IDMs unique body composition and energy requirements. Viswanathan S (20) is initiating research in this area with an ongoing study (NIH Study ID: NCT04599010), but to this point no prior studies have been done to report safety and outcomes of such a practice.

## Discussion/conclusion

**Figure 2:**
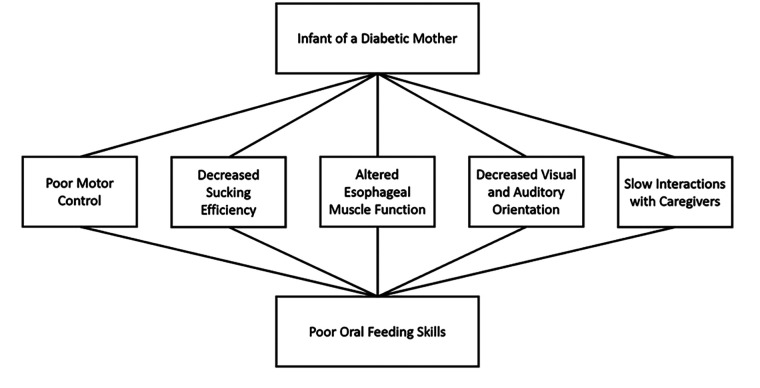
Contributing factors to poor oral feeding skills in the IDM.

Thus far, studies have shown certain groups of IDMs struggle with oral feeding. However, the exact reason for these difficulties and why all IDMs do not struggle in feeding orally during the neonatal period remain unclear. The reasons are likely multifactorial (Figure 2). What is clear is that IDMs do not behave as expected for their gestational or chronological age. The state of the IDMs is characterized as poor with slow reflexes, poor tone and motor control (10–12). A decrease in interaction with the caregiver correlates with the disinterest many IDMs seem to have in oral feeding as described by many who take care of them. Certain qualities of their oral feeding patterns are immature such as their diminished number of sucking bursts and ability to generate sucking pressure (5). The pharyngoesophageal movements of IDMs are different and slower than expected (14).

Interestingly, when IDMs are born >37 weeks GA they still have delayed onset of motor and physiological reflexes. Malkar et al. (14) and Viswanathan et al. (15) studied cohorts of IDMs that included preterm and term infants, making it more difficult to identify which gestational age of infants is more likely to encounter feeding difficulties. Moving forward, it will be important to investigate the interaction between diabetes and prematurity. Does birth at a younger gestational age lead to less exposure to a hyperglycemic and hyperinsulinemic environment *in utero* that could lessen the effects seen on the infant? Or does prematurity simply intensify the feeding challenges encountered for IDMs?

While IDMs’ behavior is reminiscent of a premature infant, the end point at which they then mature is unclear. Perhaps we as clinicians should stop labeling this behavior as premature, as it is likely a different kind of entity. Questions as to how profound and how long IDMs will experience feeding difficulties secondary to this behavior are still unanswered. Moreover, the most appropriate dietary plans and interventions to improve feeding among IDMs are largely unknown.

Limitations of the available studies discussed include the small sample size, differences in definitions of diabetic groups, lack of distinction between diabetic types, and a limited timeline of behavior assessments (<7 days of life). Inconsistencies in defining diabetic groups between studies occurs, and delineating severity remains challenging. Whether a mother is affected by type 1 vs. type 2 diabetes was also unclear in previous studies. Further investigations are needed to improve the care for these infants and provide better support to their families.

The potential for multiple complications for infants of mothers with diabetes during pregnancy such as spontaneous abortion, preterm delivery, central nervous system anomalies, congenital heart disease, or skeletal anomalies is well known in the obstetrical and neonatal fields (1). These medical conditions tend to be discussed prenatally with families, but due to the paucity of research on difficulties with oral feeding this subject tends to be left unaddressed. Unfortunately, families are ill-prepared for challenges with oral feeding after birth and become greatly frustrated when their infants struggle.

Much remains to be deciphered in the oral feeding skill progression and behavior of IDMs. Until such work is done, we continue to lack sufficient evidence-based data on which to make clinical decisions for predicting length of stay to families, determining the need for supplemental feeding devices (nasogastric tubes vs. GT), and predicting long term outcomes.

## References

[1] Blickstein I Perlman S Yenon H Shinwell ES. Pregnancy complicated by diabetes mellitus. In: Martin RJ Fanaroff AA Walsh MC, editors. Fanaroff and Martin’s Neonatal-Perinatal Medicine: Diseases of the Fetus and Infant. 11th ed. Philadelphia, PA: Elsevier (2020). p. 304–11.

[2] Gregory EC Ely DM. Trends and characteristics in gestational diabetes: united States, 2016–2020. Natl Vital Stat Rep. (2022) 71(3):1–14. 10.1016/j.ajog.2016.10.007 35877134

[3] Centers for Disease Control and Prevention. National Diabetes Statistics Report. Updated November 29, 2023. Available online at: https://www.cdc.gov/diabetes/data/statistics-report/index.html (Accessed January 11, 2024)

[4] Peng TY Ehrlich SF Crites Y Kitzmiller JL Kuzniewicz MW Hedderson MM Trends and racial and ethnic disparities in the prevalence of pregestational type 1 and type 2 diabetes in northern California: 1996–2014. Am J Obstet Gynecol. (2017) 216(2):177.e1–8. 10.1016/j.ajog.2016.10.007 27751798 PMC5290002

[5] Bromiker R Rachamim A Hammerman C Schimmel M Kaplan M Medoff-Cooper B. Immature sucking patterns in infants of mothers with diabetes. J Pediatr. (2006) 149(5):640–3. 10.1016/j.peds.2006.07.034 17095335

[6] Kiltzmiller JL Cloherty JP Younger MD Tabatabaii A Rothchild SB Epstein MF Diabetic pregnancy and perinatal morbidity. Am J Obstet Gynecol. 1978;131(5):560–80. 10.1016/0002-9378(78)90120-5 354386

[7] Browne JV Ross ES. Eating as a neurodevelopmental process for high-risk newborns. Clin Perinatol. (2011) 38(4):731–43. 10.1016/j.clp.2011.08.004 22107901

[8] Brazelton TB Cramer BG. The Earliest Relationship: Parents, Infants and the Drama of Early Attachment. 1st ed. London: Imprint Routledge (1990). Ebook published June 2019. Available online at: https://doi-org.libproxy.uthscsa.edu/10.4324/9780429481512 (Accessed February 15, 2024)

[9] Brazelton TB Nugent JK. The Neonatal Behavioral Assessment Scale. 4th ed. London: MacKeith Press (2011).

[10] Yogman MW Cole P Heidelise A Lester BM. Behavior of newborns of diabetic mothers. Infant Behav Dev. (1982) 5:331–40. 10.1016/S0163-6383(82)80043-X

[11] Rizzo T Freinkel N Metzger BE Hatcher R Burns WJ Barglow P. Correlations between antepartum maternal metabolism and newborn behavior. Am J Obstet Gynecol. (1990) 163(5 Pt 1):1458–64. 10.1016/0002-9378(90)90606-8 2240088

[12] Pressler JL Hepworth JT LaMontagne LL Sevcik RH Hesselink LF. Behavioral responses of newborns of insulin-dependent and nondiabetic, healthy mothers. Clin Nurs Res. (1999) 8(2):103–18. 10.1177/10547739922158188 10887864

[13] Botet F de Caceres ML Rosales S Costas C. Behavioral assessment of newborns from diabetic mothers. Behav Neurol. (1996) 9:1–4. 10.3233/BEN-1995-8105

[14] Malkar MB Viswanathan SK Jadcherla SR. Pilot study of pharyngoesophageal dysmotility mechanisms in dysphagic infants of diabetic mothers. Am J Perinatol. (2019) 36(12):1237–42. 10.1055/s-0038-1676632 30577057 PMC13455177

[15] Viswanathan S Batchu S Osborn E Jadcherla S. Diagnostic utility of impedance-pH monitoring in infants of diabetic mothers with oral feeding difficulties. J Perinatol. (2021) 41(8):1886–92. 10.1038/s41372-020-00832-7 32981928

[16] Viswanathan S Osborn E Jadcherla S. Body adiposity and oral feeding outcomes in infants: a pilot study. J Perinatol. (2021) 41(5):1059–64. 10.1038/s41372-021-00975-1 33597738

[17] Shekhawat PS Garland JS Shivpuri C Mick GJ Sasidharan P Pelz CJ Neonatal cord blood leptin: its relationship to birth weight, body mass index, maternal diabetes, and steroids. Pediatr Res. (1998) 43:338–43. 10.1203/00006450-199803000-00005 9505271

[18] Tapanainen P Leinonen E Ruokonen A Knip M. Leptin concentrations are elevated in newborn infants of diabetic mothers. Horm Res. (2001) 55:185–90. 10.1159/000049993 11598372

[19] Short KR Teague AM Fields DA Lyons T Chernausek SD. Lower resting energy expenditure and fat oxidation in native American and Hispanic infants born to mothers with diabetes. J Pediatr. (2015) 166(4):884–9. 10.1016/j.jpeds.2014.12.036 25648295 PMC4380761

[20] Viswanathan S. Role of Body Composition in Large for Gestational Age Infants (LGA) with Oral Feeding Difficulty. (ClinicalTrials.gov ID. NCT04599010). (2024).

